# Association of steatotic liver disease with all-cause and cardiovascular mortality among prehypertensive or hypertensive patients

**DOI:** 10.7189/jogh.15.04003

**Published:** 2025-01-17

**Authors:** Shiwei Yan, Qian Li, Wenzhe Cao, Haolong Pei, Shihan Zhen, Qingyao Wu, Xueli Yang, Fengchao Liang

**Affiliations:** 1 School of Public Health and Emergency Management, School of Medicine, Southern University of Science and Technology, Shenzhen, China; 2 Department of Occupational and Environmental Health, School of Public Health, Tianjin Medical University, Tianjin, China; 3 Key Laboratory of Prevention and Control of Major Diseases in the Population, Ministry of Education, Tianjin Medical University, Tianjin, China; 4 Tianjin Key Laboratory of Environment, Nutrition and Public Health, Tianjin Medical University, Tianjin, China

## Abstract

**Background:**

Prehypertension and hypertension often coexist with non-alcoholic fatty liver disease (NAFLD) during the progression of cardiovascular disease (CVD). International academic liver societies have recently reached a consensus to replace NAFLD with the new term ‘steatotic liver disease’ (SLD). In this study, we aimed to evaluate the impact of different SLD subtypes on all-cause and CVD mortality in individuals with prehypertension or hypertension.

**Methods:**

We included 6074 adults from the National Health and Nutrition Examination Survey (2003–18). The US fatty liver index was used as the diagnostic criterion for SLD, and participants were classified into no SLD, metabolic dysfunction-associated steatotic liver disease (MASLD), metabolic dysfunction-associated and alcohol-related liver disease (MetALD), and alcohol-related liver disease (ALD). For cases of MASLD, MetALD, and ALD, we further assessed advanced fibrosis using the fibrosis-4 (FIB-4) index. Additionally, we calculated hazard ratios (HRs) and 95% confidence intervals (CIs) using Cox proportional hazards regression models to assess the associations of SLD subtypes and advanced fibrosis with all-cause and CVD mortality.

**Results:**

There were 3505 (57.7%) participants with no SLD, 1284 (21.1%) with MASLD, 777 (12.8%) with MetALD, and 508 (8.4%) with ALD. During a median follow-up period of 8.2 years, the risk of all-cause and CVD mortality progressively increased in participants with MASLD (HR = 1.28; 95% CI = 1.01–1.63 and HR = 1.55; 95% CI = 1.04–2.33, respectively), MetALD (HR = 1.41; 95% CI = 1.05–1.88 and HR = 1.78; 95% CI = 1.10–2.87, respectively), and ALD (HR = 1.83; 95% CI = 1.32–2.53 and HR = 1.80; 95% CI = 1.01–3.19, respectively). Among the individuals with MASLD, MetALD, and ALD, advanced fibrosis was also associated with an increased risk of all-cause and CVD mortality.

**Conclusions:**

Individuals with MASLD, MetALD, and ALD had a higher risk of all-cause and CVD mortality than those without SLD. Therefore, early intervention strategies targeting SLD prevention and management may help individuals with prehypertension and hypertension to improve their long-term health.

Nowadays, non-alcoholic fatty liver disease (NAFLD) is the most common liver condition and imposes huge health and economic burdens worldwide [1]. In response to growing public health concerns and the urgent need for more accurate diagnosis, international experts have recently reached a consensus on a new nomenclature for fatty liver disease. The new term ‘steatotic liver disease’ (SLD) is classified into three subcategories: metabolic dysfunction-associated steatotic liver disease (MASLD), metabolic dysfunction-associated and alcohol-related liver disease (MetALD), and alcohol-related liver disease (ALD), based on alcohol intake and cardiometabolic risk factors (CMRFs) [2].

Mounting evidence suggests that NAFLD leads to extra-hepatic manifestations, such as metabolic diseases, cardiovascular disease (CVD), and cancers. Among these, CVD is the leading cause of death in NAFLD patients, accounting for approximately 40% of total deaths [3,4]. Subsequent population-based studies and meta-analyses found positive associations between NAFLD and structural or functional heart changes, suggesting that NAFLD could be an important predictor of cardiovascular outcomes [5,6]. However, the predictive value of SLD, according to the updated diagnostic criteria for long-term clinical outcomes, has not been sufficiently examined. Two recent studies from the Third National Health and Nutrition Examination Survey (NHANES III) investigated the impact of SLD on long-term outcomes [7,8], but there was a null association with CVD mortality.

Prehypertension, an intermediate stage between normotension and hypertension, affects 40% of the global population [9]. Among those with uncontrolled prehypertension, two-thirds are likely to develop hypertension within four years [10]. Recently, there has been growing interest in the relationship between NAFLD and blood pressure. It has been reported that NAFLD is an independent risk factor for developing prehypertension and hypertension [11–13], and approximately half of hypertensive patients have the presence of NAFLD [14]. There is evidence that suggests that the NAFLD course worsens as blood pressure elevates [15]. A large-scale prospective cohort showed that the presence of moderate/severe NAFLD may enhance the risk of cardiovascular events, particularly in prehypertensive or hypertensive people [16]. Nevertheless, population-based cohort studies with long-term follow-up exploring links of SLD and its subtypes with mortality risk in prehypertensive or hypertensive people remain scarce.

Therefore, we aimed to investigate the associations of MASLD, MetALD, ALD, and advanced fibrosis with all-cause and CVD mortality among prehypertensive or hypertensive patients, using data from NHANES 2003–18.

## METHODS

### Study population

In this study, we used data from NHANES 2003–18, which is an ongoing nationwide survey of the non-institutionalised US population using a stratified, complex, and multistage probability cluster design [17]. It was launched in the early 1960s as a survey targeting the health and nutritional status of children and adults. As of 1999, the survey has become a continuous programme, encompassing interviews (*i.e.* demographics, socioeconomic status, dietary, and health conditions) and physical examinations (*i.e.* physiological measurements and laboratory information) to meet emerging needs. It is conducted every two years on a nationally representative sample of approximately 5000 participants and is executed by the Centers for Disease Control and Prevention National Center for Health Statistics. During the period of recruitment, all participants are asked to sign informed written consent.

From eight cycles of NHANES during 2003–18, a total of 41 497 adults (20–79 years) participated in the laboratory test at the mobile examination centre. First, we excluded 30 929 participants with missing data at follow-up (n = 125) and missing information for assessing SLD and advanced fibrosis (n = 30 804). We further excluded 4494 participants with pregnancy (n = 187), viral hepatitis B or C (n = 181), and normotension (n = 4126). Finally, 6074 participants with prehypertension or hypertension were retained for the study analysis (Figure S1 in the **Online Supplementary Document**).

### Health data collection

Demographic information and other health data were obtained through questionnaire examinations, anthropometric assessments, and laboratory testing. In this study, we selected the following variables as potential confounders: age (in years), sex (men/women), race/ethnicity (Mexican American/Non-Hispanic white/Non-Hispanic black/Others), education level (less than 9th grade/9–11th grade/high school Grad/GED or equivalent/some college or AA degree/college graduate or above), annual household income in USD (<20 000/20 000–45 000/45 000–75 000/>75 000), current smoking (yes/no), body mass index (BMI) (<25 kg/m^2^/25–30 kg/m^2^/≥30 kg/m^2^), diabetes (yes/no), and hyperlipidaemia (yes/no).

### Assessment for SLD and advanced fibrosis

We adopted the US fatty liver index (FLI) to assess SLD, which has been widely used in numerous epidemiological studies [18–20]. An established US FLI cutoff of 30 was used to indicate the presence of SLD. We assessed MASLD as mild alcohol intake (<30 g/d for men or <20 g/d for women) with SLD and at least one of the following CMRFs [2]: BMI≥25 kg/m^2^ or waist circumference ≥94 cm for men or ≥80 cm for women; fasting glucose ≥100 mg/dL or glycohaemoglobin ≥5.7% or type 2 diabetes or treatment for type 2 diabetes; systolic/diastolic blood pressure ≥130/85 mm Hg or antihypertensive treatment; triglycerides ≥150 mg/dL or lipid-lowering treatment; high density lipoprotein-cholesterol (HDL-C) <40 mg/dL for men or <50 mg/dL for women or lipid-lowering treatment. We assessed MetALD as moderate alcohol intake (30–60 g/d for men or 20–50 g/d for women) with SLD and at least one CMRF. We assessed ALD as heavy alcohol intake (>60 g/d for men or >50 g/d for women), based on SLD regardless of cardiometabolic features. According to the fibrosis-4 (FIB-4) index, we classified participants with MASLD, MetALD, and ALD into two groups, including those with low to intermediate (FIB-4 ≤2.67) and high (FIB-4 >2.67) probability for advanced fibrosis (Table S1 in the **Online Supplementary Document**) [21].

### Mortality outcomes

We obtained mortality status and causes of death by linkage to the National Death Index database of the National Center for Health Statistics using a probability matching algorithm, with the death records updated to 31 December 2019. We employed the International Classification of Diseases, Tenth Revision (ICD-10) to classify mortality causes, with all-cause mortality defined as death from any cause, and CVD mortality as ICD-10 codes I00–I09, I11, I13, I20–I51, or I60–I69 [22]. We calculated the follow-up time from the date of the baseline interview to the date of death or the study end date, whichever occurred first.

### Statistical analysis

We presented continuous variables as means and standard deviations (SDs) and categorical variables as numbers and percentages. We used ANOVA and χ^2^ tests to examine the differences between groups for each SLD subtype. Hazard ratios (HRs) and 95% confidence intervals (CIs) were estimated using Cox proportional hazards regression models to evaluate associations of SLD subtypes with all-cause and CVD mortality. Covariates were included in three models: model 1, adjusted for age and sex; model 2, additionally adjusted for race, education, income, and smoking; and model 3, further adjusted for BMI, diabetes, and hyperlipidaemia. To model the dose-response relationship of alcohol intake and CMRFs, including waist circumference, fasting glucose, HDL-C, and TG, with all-cause and CVD mortality, we performed restricted cubic spline (RCS) analyses at the 10th, 50th, and 90th percentiles of the distribution, after adjusting for the same variables as in model 3. We conducted subgroup analyses to examine the association between SLD subtypes and all-cause and CVD mortality, according to sex (women or men), race (non-white or white), smoking (yes or no), BMI (<30 or ≥30), and blood pressure status (prehypertension or hypertension). We performed sensitivity analysis by adjusting additional family history of CVD and diabetes in model 3. We used R, version 4.3.2 (R Core Team, Vienna, Austria) for all statistical analyses and a two-tailed *P*-value <0.05 was considered statistically significant.

## RESULTS

### Baseline characteristics

Among the 6074 included participants, there were 3505 (57.7%) with no SLD, 1284 (21.1%) with MASLD, 777 (12.8%) with MetALD, and 508 (8.4%) with ALD (**Table 1**). Compared with the participants without SLD, those with MetALD were generally older and predominantly women, and they had lower levels of income and education, a lower proportion of current smokers, higher levels of waist circumference and DBP, and higher percentages of obesity, diabetes, and hyperlipidaemia. Levels of serum alanine aminotransferase, aspartate aminotransferase, gamma-glutamyl transferase, fasting glucose, fasting insulin, glycohemoglobin, and TG were higher in participants with MASLD, MetALD, and ALD, compared to those without SLD. Additionally, participants who died from CVD were more frequently older and men, had lower levels of income and education, higher systolic blood pressure and waist circumference, higher levels of serum gamma-glutamyl transferase, fasting glucose, glycohaemoglobin, and HDL-C (Table S2 in the **Online Supplementary Document**). Moreover, the prevalence of diabetes and hyperlipidaemia was higher among participants who died from CVD than others.

**Table 1:** Baseline characteristics of participants according to SLD subtypes

Characteristic	No SLD	MASLD	MetALD	ALD	*P*-value
No. of participants*	3505 (57.7)	1284 (21.1)	777 (12.8)	508 (8.4)	
Age (years)†	49.8 (15.9)	56.1 (14.2)	50.7 (13.9)	45.2 (13.9)	<0.001
Women*	1467 (41.9)	405 (31.5)	352 (45.3)	107 (21.1)	<0.001
Race/ethnicity*					<0.001
*Non-Hispanic white*	1526 (43.5)	670 (52.2)	356 (45.8)	191 (37.6)	
*Non-Hispanic black*	1046 (29.8)	180 (14.0)	109 (14.0)	50 (9.8)	
*Mexican American*	319 (9.1)	220 (17.1)	181 (23.3)	189 (37.2)	
*Others*	614 (17.5)	214 (16.7)	131 (16.9)	78 (15.4)	
>$75 000 annual household income*	988 (28.2)	374 (29.1)	209 (26.9)	85 (16.7)	<0.001
College graduate or above*	893 (25.5)	333 (25.9)	141 (18.2)	37 (7.3)	<0.001
Current smoker*	1130 (32.2)	235 (18.3)	201 (25.9)	218 (42.9)	<0.001
SBP (mmHg)†	130.4 (16.0)	131.3 (16.2)	130.7 (14.6)	131.2 (15.3)	0.28
DBP (mmHg)†	73.5 (12.2)	73.5 (12.9)	75.4 (12.2)	75.5 (12.2)	<0.001
Waist circumference (cm)†	95.0 (12.0)	113.9 (14.4)	113.2 (14.5)	113.7(14.4)	<0.001
BMI*					<0.001
*<25*	1248 (35.6)	46 (3.6)	30 (3.9)	17 (3.4)	
*25–30*	1406 (40.1)	332 (25.9)	191 (24.6)	130 (25.6)	
*≥30*	851 (24.3)	906 (70.6)	556 (71.6)	361 (71.1)	
ALT (U/L)†	23.0 (15.5)	31.0 (17.5)	34.7(28.1)	39.6 (27.0)	<0.001
AST (U/L)†	25.0 (24.0)	26.9 (11.0)	30.1 (23.3)	31.7 (21.5)	<0.001
GGT (U/L)†	26.0 (30.1)	42.0 (63.5)	50.0 (71.1)	64.5 (94.9)	<0.001
Fasting glucose (mg/dL)†	103.2 (26.8)	125.7 (46.2)	122.6 (43.2)	123.7 (47.8)	<0.001
Fasting insulin (pmol/L)†	47.8 (25.9)	139.3 (155.4)	138.6 (101.9)	135.9 (109.3)	<0.001
Glycohemoglobin†	5.59 (0.90)	6.20 (1.35)	6.04 (1.27)	6.06 (1.46)	<0.001
TC (mg/dL)†	197.2 (41.3)	194.0 (42.8)	202.4 (44.3)	202.4 (44.3)	<0.001
TG (mg/dL)†	114.3 (81.8)	173.5 (126.4)	176.7 (179.3)	201.6 (209.9)	<0.001
HDL-C (mg/dL)†	58.1 (17.9)	46.4 (12.1)	48.3 (12.8)	46.4 (14.3)	<0.001
Diabetes*	377 (10.8)	470 (36.6)	239 (30.8)	147 (28.9)	<0.001
Hyperlipidaemia*	1470 (41.9)	730 (56.9)	436 (56.1)	228 (44.9)	<0.001

ALD – alcohol-associated liver disease, ALT – alanine aminotransferase, AST – aspartate aminotransferase, BMI – body mass index, CI – confidence interval, CVD – cardiovascular disease, DBP – diastolic blood pressure, GGT – gamma-glutamyl transferase, HDL-C – high-density lipoprotein-cholesterol, MASLD – metabolic dysfunction-associated steatotic liver disease, MetALD – metabolic dysfunction-associated and alcohol-associated liver disease, SBP – systolic blood pressure, SLD – steatotic liver disease, TC – total cholesterol, TG – triglyceride

*Data are presented as n (%) for categorical variables; *P*-values are from χ^2^ test.

†Data are presented as mean (standard deviation) for continuous variables; *P*-values are from ANOVA.

### Association of SLD subtypes and advanced fibrosis with all-cause and CVD mortality

During a median follow-up period of 8.2 years, there were 562 all-cause deaths and 193 CVD deaths among participants with prehypertension or hypertension (**Table 2**). In the fully adjusted model (*i.e.* model 3), individuals with MASLD (HR = 1.28; 95% CI = 1.01–1.63), MetALD (HR = 1.41; 95% CI = 1.05–1.88), and ALD (HR = 1.83; 95% CI = 1.32–2.53) had a higher risk of all-cause mortality, compared with those without SLD. For CVD mortality, similar associations were observed in MASLD (HR = 1.55; 95% CI = 1.04–2.33), MetALD (HR = 1.78; 95% CI = 1.10–2.87), and ALD (HR = 1.80; 95% CI = 1.01–3.19). These results demonstrated a positive association between SLD subtypes and the risk of all-cause and CVD mortality. Advanced fibrosis was associated with an increased risk of all-cause mortality among individuals with MASLD (HR = 2.79; 95% CI = 1.60–4.85), MetALD (HR = 2.88; 95% CI = 1.62–5.11), and ALD (HR = 4.51; 95% CI = 2.27–8.97) (**Table 3**). Similarly, advanced fibrosis was also associated with an increased risk of CVD mortality among individuals with MASLD (HR = 3.70; 95% CI = 1.60–8.57), MetALD (HR = 2.91; 95% CI = 1.04–8.19), and ALD (HR = 6.45; 95% CI = 1.95–21.35). In the SLD subtype groups with advanced fibrosis as assessed by FIB-4, increases in all-cause and CVD mortality were more pronounced.

**Table 2:** Association between SLD subtypes and the risk of all-cause and CVD mortality*

	No SLD	MASLD		MetALD		ALD	
**Variables by category**	**HR (95% CI)**	**HR (95% CI)**	***P*-value**	**HR (95% CI)**	***P*-value**	**HR (95% CI)**	***P*-value**
All-cause							
*Number of death*	285	152		71		54	
*Model 1*	ref	1.10 (0.90–1.34)	0.35	1.22 (0.94–1.58)	0.14	1.84 (1.37–2.47)	<0.001
*Model 2*	ref	1.21 (0.99–1.49)	0.062	1.30 (1.00–1.70)	0.051	1.67 (1.23–2.26)	<0.001
*Model 3*	ref	1.28 (1.01–1.63)	0.044	1.41 (1.05–1.88)	0.021	1.83 (1.32–2.53)	<0.001
CVD							
*Number of death*	86	62		28		17	
*Model 1*	ref	1.45 (1.04–2.02)	0.027	1.60 (1.05–2.46)	0.031	1.97 (1.16–3.36)	0.012
*Model 2*	ref	1.71 (1.22–2.40)	0.002	1.90 (1.22–2.94)	0.004	1.88 (1.09–3.22)	0.022
*Model 3*	ref	1.55 (1.04–2.33)	0.033	1.78 (1.10–2.87)	0.018	1.80 (1.01–3.19)	0.046

ALD – alcohol-related liver disease, CI – confidence interval, CVD – cardiovascular disease, HR – hazard ratio, MASLD – metabolic dysfunction-associated steatotic alcohol-associated liver disease, MetALD – metabolic dysfunction-associated steatotic liver disease, ref – reference, SLD – steatotic liver disease

*Model 1 was adjusted for age and sex, model 2 for covariates in model 1 plus race, education, income, and smoking, and model 3 for covariates in model 2 plus BMI, diabetes, and hyperlipidaemia.

**Table 3:** Association between advanced fibrosis and the risk of all-cause and CVD mortality*

		Model 1		Model 2		Model 3	
**Variables by category**	**No. of death**	**HR (95% CI)**	***P*-value**	**HR (95% CI)**	***P*-value**	**HR (95% CI)**	***P*-value**
All-cause							
*No SLD*	285	ref		ref		ref	
*MASLD, FIB-4 ≤ 2.67*	137	1.05 (0.86–1.29)	0.64	1.16 (0.94–1.43)	0.17	1.22 (0.95–1.56)	0.12
*MASLD, FIB-4 > 2.67*	15	2.12 (1.25–3.60)	0.005	2.76 (1.62–4.70)	<0.001	2.79 (1.60–4.85)	<0.001
*MetALD, FIB-4 ≤ 2.67*	58	1.08 (0.82–1.44)	0.58	1.17 (0.88–1.56)	0.29	1.25 (0.92–1.71)	0.15
*MetALD, FIB-4 > 2.67*	13	2.70 (1.54–4.72)	<0.001	2.72 (1.55–4.79)	<0.001	2.88 (1.62–5.11)	<0.001
*ALD, FIB-4 ≤ 2.67*	45	1.60 (1.17–2.21)	0.004	1.46 (1.05–2.02)	0.023	1.61 (1.14–2.28)	0.007
*ALD, FIB-4 > 2.67*	9	6.17 (3.16–12.03)	<0.001	5.20 (2.64–10.22)	<0.001	4.51 (2.27–8.97)	<0.001
CVD							
*No SLD*	86	ref		ref		ref	
*MASLD, FIB-4 ≤ 2.67*	55	1.37 (0.98–1.93)	0.07	1.61 (1.14–2.29)	0.007	1.47 (0.98–2.22)	0.065
*MASLD, FIB-4 > 2.67*	7	3.03 (1.38–6.64)	0.006	4.30 (1.93–9.57)	<0.001	3.70 (1.60–8.57)	0.002
*MetALD, FIB-4 ≤ 2.67*	24	1.50 (0.95–2.36)	0.079	1.78 (1.12–2.84)	0.015	1.68 (1.02–2.77)	0.043
*MetALD, FIB-4 > 2.67*	4	2.65 (0.97–7.27)	0.058	3.13 (1.13–8.68)	0.028	2.91 (1.04–8.19)	0.043
*ALD, FIB-4 ≤ 2.67*	14	1.70 (0.96–3.03)	0.069	1.61 (0.90–2.89)	0.11	1.55 (0.84–2.86)	0.17
*ALD, FIB-4 > 2.67*	3	6.98 (2.19–22.24)	0.001	7.52 (2.32–24.43)	<0.001	6.45 (1.95–21.35)	0.002

ALD – alcohol-related liver disease, CI – confidence interval, CVD – cardiovascular disease, FIB-4 – fibrosis-4, HR – hazard ratio, MASLD – metabolic dysfunction-associated steatotic liver disease, MetALD – metabolic dysfunction-associated and alcohol-related liver disease, ref – reference, SLD – steatotic liver disease

*Model 1 was adjusted for age and sex, model 2 for covariates in model 1 plus race, education, income, and smoking, and model 3 for the covariates in model 2 plus BMI, diabetes, and hyperlipidaemia.

**Figure 1:**
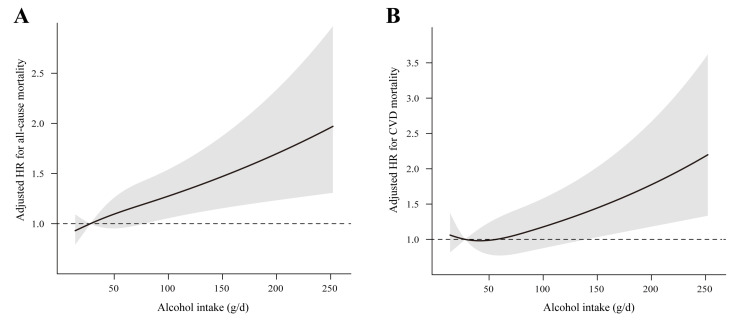
Restricted cubic splines for the association of alcohol intake with all-cause and CVD mortality in SLD individuals. Adjustments included age, sex, race, education, income, smoking, BMI, diabetes, and hyperlipidaemia. **Panel A.** All-cause mortality. **Panel B.** CVD mortality. CVD – cardiovascular disease, SLD – steatotic liver disease

**Figure 2:**
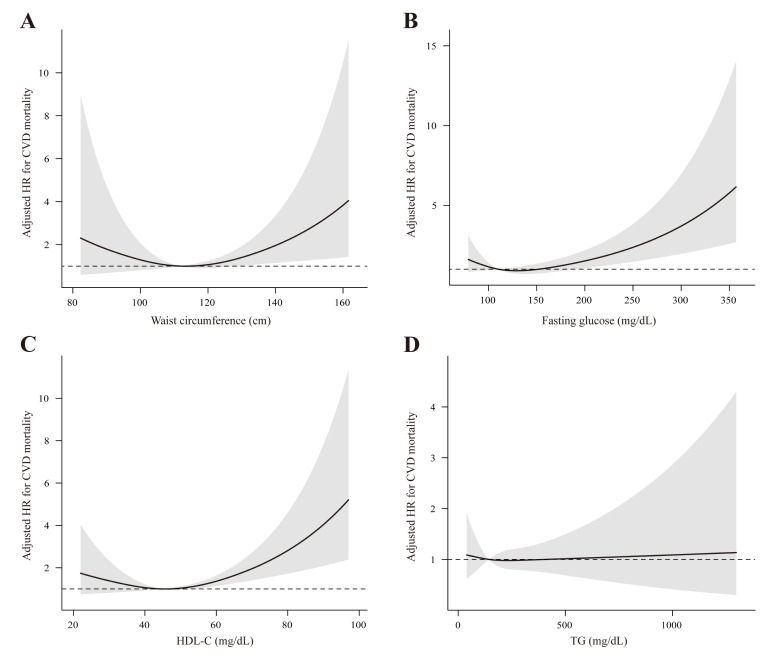
Restricted cubic splines for the association of waist circumference, fasting glucose, HDL-C, and TG with CVD mortality in SLD individuals. Adjustments included age, sex, race, education, income, smoking, BMI, diabetes, and hyperlipidaemia. **Panel A.** Waist circumference. **Panel B.** Fasting glucose. **Panel C.** HDL-C. **Panel D.** TG. CVD – cardiovascular disease, HDL-C – high-density lipoprotein-cholesterol, SLD – steatotic liver disease, TG – triglycerides.

Subsequently, we adopted RCS analyses to model the dose-response relationship of alcohol intake and CMRFs with all-cause and CVD mortality (**Figure 1**). There were nonlinear associations between waist circumference (*P* = 0.043), fasting glucose (*P* = 0.037), and HDL-C (*P* = 0.012) (**Figure 2**, Panels A–C). Our RCS analyses presented a J-shaped association of fasting glucose with CVD mortality and U-shaped associations between waist circumference and HDL-C and CVD mortality, with risk increasing above 115.4 cm for waist circumference and 46.5 mg/dL for HDL-C. Additionally, we conducted RCS analyses for the association between CMRFs and all-cause mortality (Figure S2 in the **Online Supplementary Document**).

### Subgroup and sensitivity analysis

We conducted the subgroup analyses according to sex, race, smoking, BMI, and blood pressure status, and found that men, white, non-current smokers, obese, and hypertensive individuals were more influenced by ALD regarding a risk of all-cause mortality. MetALD was associated with an increased risk of all-cause mortality, especially in non-current smokers and hypertensive patients. For MASLD, similar results were observed in white individuals and non-current smokers (Table S3 in the **Online Supplementary Document**). Moreover, the risky effect of MASLD and MetALD on CVD mortality was more prominent in women, non-current smokers, and hypertensive individuals. Additionally, ALD was associated with an increased risk of CVD mortality only in hypertensive patients (Table S4 in the **Online Supplementary Document**). Furthermore, there was no significant interaction between SLD subtypes and all-cause and CVD mortality. The sensitivity analysis revealed that further adjustment for a family history of CVD and diabetes did not change the statistical significance of the initial results, suggesting relatively robust associations between SLD subtypes and advanced fibrosis with mortality outcomes (Table S5 in the **Online Supplementary Document**).

## DISCUSSION

In this prospective study, we analysed data from the NHANES 2003–18 to evaluate the effect of SLD on mortality among individuals with prehypertension or hypertension. Our results showed that MASLD, MetALD, and ALD increased the risks of all-cause and CVD mortality, and these associations were more prominent in advanced fibrosis.

### Comparison with previous studies

Since the new term of SLD was proposed, several epidemiological studies have investigated the impact of SLD and its subtypes on long-term outcomes. Two recent studies with long follow-up periods found that MetALD was associated with an increased risk of all-cause mortality, but no such increase emerged with CVD mortality [7,8]. Compared with these studies, the current evaluation of mortality with SLD showed some discrepancies. This could be due to the following reasons. First, the two published works assessed SLD using ultrasonography, but we employed the assessment of SLD by an algorithm based on metabolic biomarkers, which appeared to have a closer link with prognostic outcomes in contrast to ultrasonography and to be measured more easily in large-scale population-based studies. Second, this study was conducted among adults with prehypertension or hypertension. Considering that elevated blood pressure is a significant pathogenic factor for CVD, individuals with prehypertension or hypertension may contribute to the occurrence of CVD and even death in contrast to the general population. Furthermore, a large-scale study from the Korea National Health Insurance Service found a positive association between SLD subtypes and the incidence of CVD [23], similar to our findings, which showed that the risk of CVD mortality gradually increased from MASLD to MetALD and further to ALD. These insights are crucial for identifying SLD subtypes that may help prevent cardiovascular events.

As a heterogeneous disorder, SLD encompasses a spectrum of pathological conditions, in which advanced fibrosis has a deterioration course with its characteristic of hepatocyte injury. The FIB-4 is widely used to identify advanced fibrosis due to its validated predictive value in studies of histologically confirmed NAFLD [24–26]. A recent study from the Korea NHANES (2007–15) with 46 456 participants showed that the FIB-4 was a significant predictor of mortality, particularly from all-cause, cardiovascular, and liver-related diseases [27]. Our observations indicated that advanced fibrosis within each SLD subtype may serve as a marker to distinguish between low- and high-risk SLD, associated with a higher risk of all-cause and CVD mortality. Nevertheless, given the limited understanding of SLD and its progression, more attention should be paid to the development of advanced fibrosis and its subsequent health impacts.

Individuals with MetALD in our study had a higher risk of CVD mortality than those with MASLD, indicating that more alcohol intake was likely to promote the development of CVD when combined with CMRFs. Before the new criteria for SLD were proposed, NAFLD and ALD were generally considered distinct diseases and were studied independently. Nevertheless, growing evidence suggests that alcohol and metabolic disorders coexisting in SLD exert promotional or synergistic effects due to their overlapping and shared pathogenic factors. Metabolic perturbations caused by increased adiposity and insulin resistance contribute to hepatocytic lipid accumulation [28]. Additionally, alcohol can accelerate lipid accumulation via the reduction of mitochondrial β-oxidation of fatty acids and very low-density lipoprotein secretion in the liver [29]. Importantly, these processes, which worsen hepatic steatosis, can lead to vascular injury due to metabolic disturbances and alcohol intake, thereby contributing to the development of CVD [30,31].

In the context of SLD, we observed a J-shaped relationship between alcohol intake and CVD mortality, suggesting that higher alcohol intake is linked to a greater risk of CVD mortality. This phenomenon has been recently replicated in the Korea National Health Insurance Service cohort, exhibiting a similar rise in the risk of CVD [23]. Also, certain CMRFs were associated with CVD mortality in a positive dose response. Especially, an increased risk of CVD mortality was significant in waist circumference and fasting glucose, providing epidemiological evidence that adiposity and insulin resistance play critical roles in the progression of CVD in individuals with SLD.

### Mechanistic interpretation

The biological mechanisms underlying the link between SLD and CVD remain unclear, but plausible interpretations from experiments and clinical studies may be suggested. Recently, increasing attention has been paid to epigenetic regulation, with micro-RNAs being key regulators of lipid transport and synthesis [32,33]. A main component of the hepatic micro-RNA pool, miR-122, was found to have relatively low expression levels in NAFLD/NASH humans [34,35]. Given its role in regulating lipid and cholesterol metabolism, miR-122 may be involved in pathways related to cardiovascular modulation [36]. Also, there are common pathophysiologic mechanisms between hepatic steatosis and high blood pressure. In the case of NAFLD, lipid metabolism triggers an innate immune response and produces pro-inflammatory factors that have been confirmed to activate the renin-angiotensin system and sympathetic nervous system with a high correlation [37–41]. This may be a critical pathogenic process in prehypertension and hypertension. Since explanations regarding the link between hepatic steatosis and CVD are mainly based on the concept of NAFLD, future research should further probe the potential mechanisms by which alcohol causes adverse cardiovascular outcomes in prehypertensive or hypertensive individuals with CMRFs.

### Strengths and limitations

The strengths of this study include its longitudinal design, coverage of multi-ethnic participants from NHANES, and the fact that it is the first to explore the association of SLD subtypes with all-cause and CVD mortality among individuals with prehypertension or hypertension. Additionally, we drew robust conclusions across different subgroups and performed sensitivity analysis. However, several limitations should be noted. First, SLD was assessed using non-invasive scores, which led to the exclusion of numerous cases due to missing data. Although the cases might be underestimated probably due to random missing data, this study still observed the significant associations of mortality with SLD and its subtypes. In future studies, more attention should be paid to differences in prognostic risk of SLD between liver ultrasound and non-invasive assessments. Second, alcohol intake was based on self-reports from participants, which could introduce recall bias to increase the possibility of misclassification in SLD subtypes. Despite this, we should note that self-reported alcohol intake is considered a reliable and valid method [42]. Third, although a set of confounding variables was included in the model, we are unable to ensure the potential impacts of additional unmeasured and residual confounders. Additionally, the relatively small number of death cases among participants with a FIB-4 > 2.67 may have limited our ability to fully evaluate the association between advanced fibrosis and mortality risk. Lastly, due to the observational nature of the study, it is difficult to establish causality. Future research, such as Mendelian randomisation studies or randomised controlled trials, is necessary to confirm the causal relationship between SLD subtypes and mortality outcomes.

## CONCLUSIONS

Our results showed that SLD subtypes and advanced fibrosis had risk effects on mortality among participants with prehypertension or hypertension. Specifically, a higher risk of all-cause and CVD mortality was found in MetALD in contrast to MASLD, indicating the key role of alcohol intake in conjunction with CMRFs in adverse health outcomes. These findings provide a basis for future research into SLD in risk prediction and highlight that early intervention strategies targeting SLD prevention and management may improve long-term health in high-risk populations.

## Additional material


Online Supplementary Document
