# Supplementary material for: Association of steatotic liver disease with all-cause and cardiovascular mortality among prehypertensive or hypertensive patients
Source: J Glob Health. 2025 Jan 17;15:04003. doi: 10.7189/jogh.15.04003 (PMC11737813; doi:10.7189/jogh.15.04003)
Supplement: Online Supplementary Document [file jogh-15-04003-s001.pdf]

Supplement to: Yan S, Li Q, Cao W, Pei H, Zhen S, Wu Q, Yang X, Liang F. Association of steatotic liver disease with all-cause and cardiovascular mortality among prehypertensive or hypertensive patients. J Glob Health. 2025;15:04003.

Table S1. Computational methods for non-invasive assessment models of liver disease and other disease definitions 1
Table S2. Baseline characteristics of study variables by CVD mortality status 2
Table S3. Subgroup analyses of the associations between SLD subtypes and all-cause mortality .. 3
Table S4. Subgroup analyses of the associations between SLD subtypes and CVD mortality 4
Table S5. Sensitivity analyses: Association of SLD subtypes and advanced fibrosis with the risk of all-cause and CVD mortality 5
Figure S1. Flowchart of the screening process of eligible participants from NHANES 2003-18.... 6
Figure S2. Restricted cubic splines for the association of waist circumference, fasting glucose, HDL-C, and TG with all-cause mortality in SLD individuals 7

Table S1. Computational methods for non-invasive assessment models of liver disease and other disease definitions

Scoring system	Algorithms
US FLI [1]	$\frac{e^{-0.8073 \times \text{non-Hispanic black} + 0.3458 \times \text{Mexican American} + 0.0093 \times \text{age} + 0.6151 \times \ln(\text{GGT}) + 0.0249 \times \text{waist circumference} + 1.1792 \times \ln(\text{insulin}) + 0.8242 \times \ln(\text{glucose}) - 14.7812}}{1 + e^{-0.8073 \times \text{non-Hispanic black} + 0.3458 \times \text{Mexican American} + 0.0093 \times \text{age} + 0.6151 \times \ln(\text{GGT}) + 0.0249 \times \text{waist circumference} + 1.1792 \times \ln(\text{insulin}) + 0.8242 \times \ln(\text{glucose}) - 14.7812}} \times 100$
FIB-4 [2]	[age (year) × AST (U/L)] / [platelet (10^9/L) × ALT (U/L)$^{1/2}$]
**Disease**	**Definition**
Prehypertension [3]	SBP of 120-140 mmHg and/or DBP of 80-90 mmHg and without taking anti-hypertensive medication or established hypertension
Hypertension [3]	Self-reported hypertension, SBP ≥140 mmHg or DBP ≥90 mmHg or use of anti-hypertensive medication
Diabetes [4]	Self-reported diagnosis, fasting glucose ≥126 mg/dL, glycohemoglobin ≥6.5%, use of insulin or oral hypoglycemic medication
Hyperlipidaemia	Self-reported diagnosis, TC >240 mg/dL or taking cholesterol-lowering medication

ALT – alanine aminotransferase, AST – aspartate aminotransferase, DBP – diastolic blood pressure, GGT – gamma glutamyl transferase, HDL-C – high-density lipoprotein-cholestero, SBP – systolic blood pressure, TC – total cholesterol

REFERENCES

1 Ruhl CE, Everhart JE. Fatty liver indices in the multiethnic United States National Health and Nutrition Examination Survey. Aliment Pharmacol Ther. 2015;41(1):65–76.

2 Shah AG, Lydecker A, Murray K, Tetri BN, Contos MJ, Sanyal AJ. Nash Clinical Research N. Comparison of noninvasive markers of fibrosis in patients with nonalcoholic fatty liver disease. Clin Gastroenterol Hepatol. 2009; 7:1104–1112.

3 Chobanian AV, Bakris GL, Black HR, Cushman WC, Green LA, Izzo JL Jr, et al. The Seventh Report of the Joint National Committee on Prevention, Detection, Evaluation, and Treatment of High Blood Pressure: the JNC 7 report. JAMA. 2003;289(19):2560–72.

4 Zou X, Zhou X, Zhu Z, Ji L. Novel subgroups of patients with adult-onset Diabetes in Chinese and US populations. The Lancet Diabetes Endocrinology. 2019;7(1):9–11.

Table S2. Baseline characteristics of study variables by CVD mortality status

Characteristic	Death from CVD	Other participants	*P*-value
No. of participants*	193 (3.2)	5881 (96.8)	
Age (years)†	64.7 (10.9)	50.4 (15.4)	<0.001
Women*	58 (30.1)	2273 (38.7)	0.016
Race/ethnicity*			0.024
Non-Hispanic white	96 (49.7)	2647 (45.0)	
Non-Hispanic black	54 (28.0)	1331 (22.6)	
Mexican American	22 (11.4)	887 (15.1)	
Others	21 (10.9)	1016 (17.3)	
>$75 000 annual household income*	18 (9.3)	1638 (27.9)	<0.001
College graduate or above*	25 (13.0)	1379 (23.5)	<0.001
Current smoker*	65 (33.7)	1719 (29.2)	0.40
SBP (mmHg)†	136.8 (21.0)	130.5 (15.6)	<0.001
DBP (mmHg)†	70.3 (13.9)	74.0 (12.3)	<0.001
Waist circumference (cm)†	106.6 (16.6)	102.8 (16.0)	0.001
BMI*			0.84
<25	41 (21.2)	1300 (22.1)	
25–30	63 (32.6)	1996 (33.9)	
≥30	89 (46.1)	2585 (44.0)	
ALT (U/L)†	26.9 (31.3)	27.6 (19.5)	0.65
AST (U/L)†	28.4 (25.3)	26.6 (21.6)	0.24
GGT (U/L)†	48.9 (80.2)	35.2 (53.0)	<0.001
Fasting glucose (mg/dL)†	128.2 (61.8)	111.6 (36.3)	<0.001
Fasting insulin (pmol/L)†	83.8 (67.3)	86.2 (100.0)	0.74
Glycohemoglobin†	6.35 (1.77)	5.80 (1.11)	<0.001
TC (mg/dL)†	199.8 (45.0)	197.6 (42.3)	0.46
TG (mg/dL)†	155.7 (112.7)	141.6 (127.4)	0.13
HDL-C (mg/dL)†	56.4 (18.8)	53.3 (16.8)	0.011
Diabetes*	64 (33.2)	1169 (19.9)	<0.001
Hyperlipidaemia*	107 (55.4)	2757 (46.9)	0.019

ALT – alanine aminotransferase, AST – aspartate aminotransferase, BMI – body mass index, CI – confidence interval, CVD – cardiovascular disease, DBP – diastolic blood pressure, GGT – gamma glutamyl transferase, HDL-C – high-density lipoprotein-cholestero, SBP – systolic blood pressure, TC – total cholesterol, TG – triglycerides

*Data are presented as n (%) for categorical variables; *P*-values are from χ^2 test.

†Data are presented as mean (standard deviations) for continuous variables; *P*-values are from ANOVA.

Table S3. Subgroup analyses of the associations between SLD subtypes and all-cause mortality

	No SLD	MASLD	MetALD	ALD	*P* for interaction
	HR (95% CI)	HR (95% CI)	HR (95% CI)	HR (95% CI)	
Sex					0.84
Men	1.00 (ref)	1.32 (0.98-1.77)	1.37 (0.95-1.98)	1.84 (1.27-2.65)	
Women	1.00 (ref)	1.22 (0.78-1.90)	1.40 (0.87-2.27)	1.70 (0.78-3.72)	
Race					0.062
White	1.00 (ref)	1.43 (1.05-1.94)	1.23 (0.83-1.83)	1.69 (1.05-2.69)	
Non-white	1.00 (ref)	0.89 (0.60-1.32)	1.42 (0.94-2.14)	1.62 (1.04-2.52)	
Smoking					0.21
Yes	1.00 (ref)	0.96 (0.60-1.54)	0.95 (0.57-1.58)	1.47 (0.92-2.35)	
No	1.00 (ref)	1.46 (1.09-1.95)	1.78 (1.25-2.54)	2.24 (1.39-3.59)	
BMI, kg/m^2					0.45
<30	1.00 (ref)	1.03 (0.75-1.43)	1.16 (0.77-1.73)	1.73 (1.14-2.63)	
≥30	1.00 (ref)	1.37 (0.92-2.04)	1.56 (0.99-2.46)	1.81 (1.07-3.09)	
Blood pressure					0.76
Prehypertension	1.00 (ref)	1.46 (0.81-2.64)	1.31 (0.64-2.67)	2.22 (0.99-4.97)	
Hypertension	1.00 (ref)	1.23 (0.94-1.60)	1.43 (1.04-1.98)	1.67 (1.17-2.40)	

ALD – alcohol-related liver disease, BMI – body mass index, CI – confidence interval, CVD – cardiovascular disease, HR – hazard ratio, MASLD – metabolic dysfunction-associated steatotic liver disease, MetALD – metabolic dysfunction-associated and alcohol-related liver disease, ref – reference, SLD – steatotic liver disease

The strata variable was not included in the model when stratifying by itself.

Table S4. Subgroup analyses of the associations between SLD subtypes and CVD mortality

	No SLD	MASLD	MetALD	ALD	*P* for interaction
	HR (95% CI)	HR (95% CI)	HR (95% CI)	HR (95% CI)	
Sex					0.29
Men	1.00 (ref)	1.38 (0.84-2.26)	1.19 (0.62-2.26)	1.66 (0.87-3.16)	
Women	1.00 (ref)	2.16 (1.04-4.49)	2.93 (1.37-6.26)	1.48 (0.31-6.96)	
Race					0.053
White	1.00 (ref)	1.64 (0.94-2.88)	1.15 (0.55-2.43)	1.35 (0.53-3.45)	
Non-white	1.00 (ref)	0.96 (0.52-1.76)	1.78 (0.97-3.27)	1.54 (0.75-3.17)	
Smoking					0.60
Yes	1.00 (ref)	1.13 (0.50-2.58)	1.79 (0.80-4.00)	1.83 (0.82-4.07)	
No	1.00 (ref)	1.66 (1.03-2.68)	1.88 (1.03-3.45)	1.81 (0.73-4.52)	
BMI, kg/m^2					0.75
<30	1.00 (ref)	1.13 (0.63-2.04)	1.71 (0.90-3.28)	1.72 (0.81-3.68)	
≥30	1.00 (ref)	1.57 (0.86-2.87)	1.66 (0.82-3.38)	1.81 (0.76-4.33)	
Blood pressure					0.49
Prehypertension	1.00 (ref)	0.77 (0.21-2.86)	1.12 (0.28-4.47)	0.78 (0.09-6.88)	
Hypertension	1.00 (ref)	1.68 (1.09-2.61)	1.91 (1.14-3.20)	1.85 (1.01-3.40)	

ALD – alcohol-related liver disease, BMI – body mass index, CI – confidence interval, CVD – cardiovascular disease, HR – hazard ratio, MASLD – metabolic dysfunction-associated steatotic liver disease, MetALD – metabolic dysfunction-associated and alcohol-related liver disease, ref – reference, SLD – steatotic liver disease

The strata variable was not included in the model when stratifying by itself.

Table S5. Sensitivity analyses: Association of SLD subtypes and advanced fibrosis with the risk of all-cause and CVD mortality

	All-cause		CVD	
	HR (95% CI)	*P*-value	HR (95% CI)	*P*-value
SLD subtypes				
No SLD	1.00 (ref)		1.00 (ref)	
MASLD	1.28 (1.01-1.63)	0.044	1.56 (1.04-2.34)	0.030
MetALD	1.41 (1.06-1.88)	0.020	1.78 (1.10-2.88)	0.018
ALD	1.85 (1.33-2.56)	<0.001	1.83 (1.02-3.27)	0.043
Advanced fibrosis				
No SLD	1.00 (ref)		1.00 (ref)	
MASLD, FIB-4 ≤2.67	1.22 (0.95-1.56)	0.12	1.48 (0.98-2.23)	0.061
MASLD, FIB-4 >2.67	2.80 (1.61-4.89)	<0.001	3.86 (1.66-8.99)	0.002
MetALD, FIB-4 ≤2.67	1.26 (0.92-1.71)	0.15	1.67 (1.01-2.76)	0.045
MetALD, FIB-4 >2.67	2.87 (1.62-5.09)	<0.001	2.99 (1.06-8.43)	0.038
ALD, FIB-4 ≤2.67	1.63 (1.15-2.32)	0.006	1.57 (0.84-2.93)	0.16
ALD, FIB-4 >2.67	4.52 (2.27-9.00)	<0.001	6.91 (2.09-22.91)	0.002

ALD – alcohol-related liver disease, CI – confidence interval, CVD – cardiovascular disease, FIB-4 – fibrosis-4 index, HR – hazard ratio, MASLD – metabolic dysfunction-associated steatotic liver disease, MetALD – metabolic dysfunction-associated and alcohol-related liver disease, ref – reference, SLD – steatotic liver disease

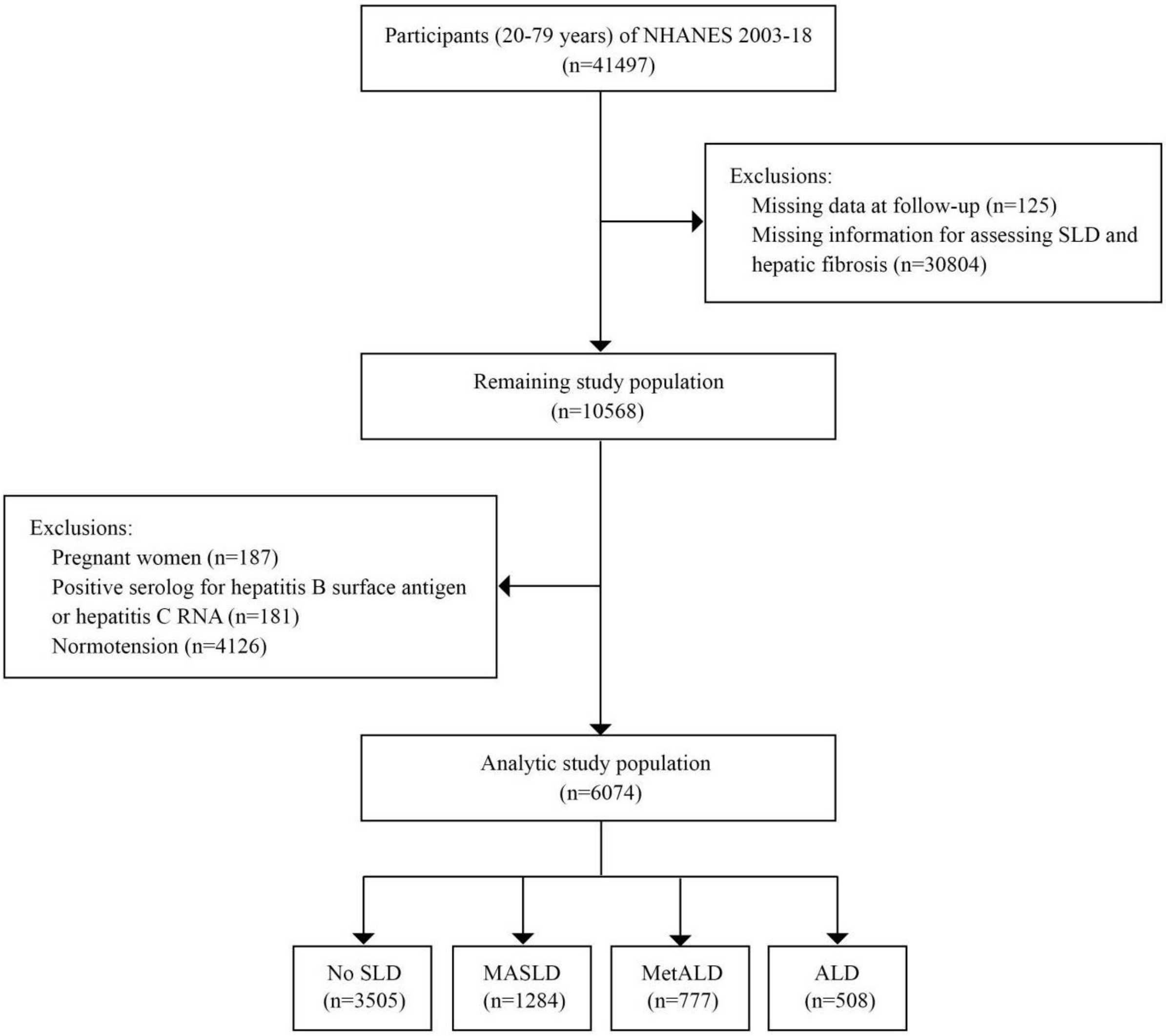

Figure S1. Flowchart of the screening process of eligible participants from NHANES 2003-18

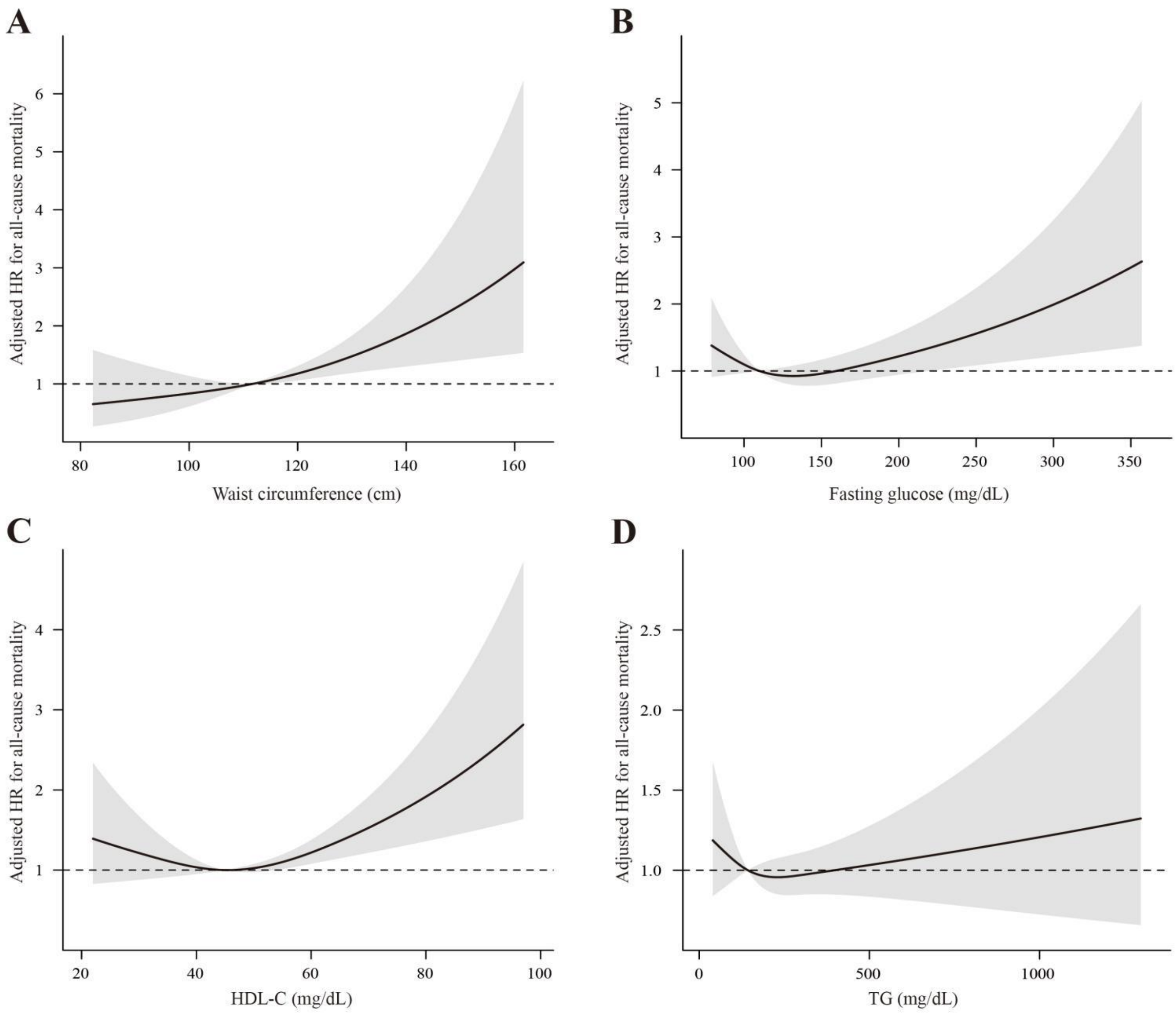

Figure S2. Restricted cubic splines for the association of waist circumference, fasting glucose, HDL-C, and TG with all-cause mortality in SLD individuals. Adjustments included age, sex, race, education, income, smoking, BMI, diabetes, and hyperlipidaemia. **Panel A.** Waist circumference. **Panel B.** Fasting glucose. **Panel C.** HDL-C. **Panel D.** TG. CVD – cardiovascular disease, HDL-C – high-density lipoprotein-cholesterol, SLD – steatotic liver disease, TG – triglycerides.
